# Correction to: Disability inclusion in national surveys

**DOI:** 10.1093/haschl/qxae145

**Published:** 2024-12-09

This is a correction to: Caroline Cerilli, Varshini Varadaraj, Jennifer Choi, Fiona Sweeney, Franz Castro, Scott D Landes, Bonnielin K Swenor, Disability inclusion in national surveys, *Health Affairs Scholar*, Volume 2, Issue 9, September 2024, qxae117, https://doi.org/10.1093/haschl/qxae117

The authors regret that the initial publication of this paper erroneously attributed the administrator for surveys included in Table 1 to the Centers for Disease Control and Prevention (CDC). In this revised version, references to the CDC have been removed from the following sections: Abstract, Introduction (final sentence), Methods (Survey Selection section), Results, and Discussion (Limitations section). The text now clarifies that while the surveys included in these analyses were sourced from the CDC Q-Bank, which compiles research surveys from various federal agencies, they are actually administered or co-administered by the US Department of Health and Human Services (HHS) and/or the US Census Bureau.

To enhance clarity the following additional changes have been made:

- The Survey Selection section of the Methods now includes the following language:

“List of All Surveys and Programs’ page (https://www.census.gov/programs-surveys/surveys-programs.html) and/or the CDC-s Q-Bank (https://wwwn.cdc.gov/QBank/Search/Surveys.aspx#/Surveys). The CDC Q-Bank is a resource for researchers to find validated research questions used in federal surveys and includes surveys from various agencies and offices. These sites were used to identify surveys for inclusion screening as they provide a comprehensive list of U.S. data on health equity and social determinants of health across sectors.”

- The Survey Methodology Analysis section of the Methods now includes the following language:

“Data collection methods used for survey screening, primary sampling, participant assistance, and longitudinal follow up were included. Data collection methods used exclusively for survey nonresponse follow up were not included.”

The description of publicly available data used for analyses was updated with the most current information in the Results section.

- The sentence “Thirteen surveys collected data via online forms, 10 by paper, 16 in person, and 18 over the telephone.“ now reads “Thirteen surveys collected data via online forms, 11 by paper, 15 in person, and 20 over the telephone.” Figure 1 also reflects these changes to the data.
- The last column in Table 1, indicating having more than one data collection modality, now indicates ’no’ for the Principal Follow-Up Survey and ‘yes’ for the National Health Interview Survey.

